# Whole genome variant association across 100 dogs identifies a frame shift mutation in *DISHEVELLED 2* which contributes to Robinow-like syndrome in Bulldogs and related screw tail dog breeds

**DOI:** 10.1371/journal.pgen.1007850

**Published:** 2018-12-06

**Authors:** Tamer A. Mansour, Katherine Lucot, Sara E. Konopelski, Peter J. Dickinson, Beverly K. Sturges, Karen L. Vernau, Shannon Choi, Joshua A. Stern, Sara M. Thomasy, Sophie Döring, Frank J. M. Verstraete, Eric G. Johnson, Daniel York, Robert B. Rebhun, Hsin-Yi Henry Ho, C. Titus Brown, Danika L. Bannasch

**Affiliations:** 1 Department of Population Health and Reproduction, School of Veterinary Medicine, University of California Davis, Davis, CA, United States of America; 2 Department of Clinical Pathology, School of Medicine, University of Mansoura, Mansoura Egypt; 3 Integrative Genetics and Genomics Graduate Group, University of California Davis, Davis, CA, United States of America; 4 Department of Cell Biology and Human Anatomy, School of Medicine, University of California Davis, Davis, CA, United States of America; 5 Department of Surgical and Radiological Sciences, School of Veterinary Medicine, University of California Davis, Davis, CA, United States of America; 6 Department of Medicine and Epidemiology, School of Veterinary Medicine, University of California Davis, Davis, CA, United States of America; 7 William R. Pritchard Veterinary Medical Teaching Hospital, School of Veterinary Medicine, University of California Davis, Davis, CA, United States of America; 8 Genome Center, University of California Davis, Davis, CA, United States of America; University of Bern, SWITZERLAND

## Abstract

Domestic dog breeds exhibit remarkable morphological variations that result from centuries of artificial selection and breeding. Identifying the genetic changes that contribute to these variations could provide critical insights into the molecular basis of tissue and organismal morphogenesis. Bulldogs, French Bulldogs and Boston Terriers share many morphological and disease-predisposition traits, including brachycephalic skull morphology, widely set eyes and short stature. Unlike other brachycephalic dogs, these breeds also exhibit vertebral malformations that result in a truncated, kinked tail (screw tail). Whole genome sequencing of 100 dogs from 21 breeds identified 12.4 million bi-allelic variants that met inclusion criteria. Whole Genome Association of these variants with the breed defining phenotype of screw tail was performed using 10 cases and 84 controls and identified a frameshift mutation in the WNT pathway gene *DISHEVELLED 2* (*DVL2)* (Chr5: 32195043_32195044del, *p* = 4.37 X 10^−37^) as the most strongly associated variant in the canine genome. This *DVL2* variant was fixed in Bulldogs and French Bulldogs and had a high allele frequency (0.94) in Boston Terriers. The *DVL2* variant segregated with thoracic and caudal vertebral column malformations in a recessive manner with incomplete and variable penetrance for thoracic vertebral malformations between different breeds. Importantly, analogous frameshift mutations in the human *DVL1* and *DVL3* genes cause Robinow syndrome, a congenital disorder characterized by similar craniofacial, limb and vertebral malformations. Analysis of the canine DVL2 variant protein showed that its ability to undergo WNT-induced phosphorylation is reduced, suggesting that altered WNT signaling may contribute to the Robinow-like syndrome in the screwtail breeds.

## Author summary

Some dog breeds are characterized by extreme morphological differences from their ancestor, the wolf. One group of three breeds (Bulldog, French Bulldog and Boston Terrier) is characterized by a wide head, short muzzle, widely spaced eyes, small size and abnormalities of the vertebral bones of the back and tail. These breeds are referred to as the screw tail breeds since the characteristic that is unique and easy to see in these breeds is their shortened and kinked tails. These breed have become increasingly popular as pet dogs, although they have health issues associated with their morphology. We analyzed the genome sequences of 100 dogs, including 10 screw tail dogs, and identified all the genetic differences between those dogs. We then compared these differences to identify changes in the DNA sequences associated with screw tail. The mutation and the affected gene identified are very similar to the types of mutations that have been shown to be responsible for a rare human disorder with similar clinical abnormalities, called Robinow syndrome. We demonstrate that the dog mutation makes an altered protein that affects an important cell-cell communication system crucial for tissue development.

## Introduction

Morphological differences have been one of the primary drivers of dog breed formation since wolf domestication and subsequent selection to create dog breeds [1]. In many cases, the morphological traits are also genetically linked to disease-predisposition traits [2–6]. Therefore, it is of great interest to determine the breed specific genetic variations, as this knowledge could provide insights into not only the evolution of dog breeds, but also the mechanisms of tissue morphogenesis and disease pathogenesis.

Some subsets of dog breeds share distinctive morphologies. A shortened and kinked tail—which is referred to as a “screw tail”—is one of the distinctive morphological traits that characterizes Bulldogs, French Bulldogs and Boston Terriers, which were historically developed from the Bulldog breed and, thus, closely related [7]. These breeds also share a craniofacial morphological phenotype referred to as brachycephaly, which includes both profound shortening of the muzzle and widening of the skull. Possibly due to the width of their heads, these three breeds have the highest rate of cesarean section among dog breeds [8]. The three breeds are also all relatively short in stature being less than 17 inches tall at the shoulder. In addition to morphological features, certain diseases are found at a high prevalence across all 3 breeds such as vertebral malformations [9–13], cleft lip and cleft palate [14–16], congenital heart disease [17, 18, 19], and glioma [5, 20, 21].

Molecular characterization of the cause of short tails in dogs demonstrated that a synonymous mutation in the T box transcription factor gene confers a short tail (bob tail) phenotype in the Pembroke Welsh Corgi breed [22]. Although this mutation is common across a wide range of dog breeds, it is not responsible for the short tail seen in Bulldogs and Boston Terriers [23]. Screw tail is distinguishable from the short tail phenotypes in other breeds due to presence of vertebral malformations and fusion of the caudal vertebrae in addition to absence of caudal vertebrae. Additional conformational sequelae of vertebral malformation in screw tail breeds includes kyphosis, lordosis or scoliosis most commonly affecting the thoracic vertebral column, and typically resulting from wedge, hemi-vertebrae and butterfly vertebrae [24, 25]. Although vertebral malformations affecting thoracic vertebrae in screw tail breeds are common, they are rarely directly associated with clinical signs [9]. However, it may increase their risk of developing other diseases such as intervertebral disc disease, to which the French Bulldog and Boston Terrier are predisposed [6, 26].

Brachycephalic skull morphology has been previously investigated in dogs and has a complex etiology. Multiple different chromosomal regions are associated and may differ between breeds. In a Genome Wide Association (GWAS) for skull shape across dog breeds, significant associations were identified on CFA (*Canis familiaris*) 1, CFA5, CFA24, CFA30 and CFA32 for brachycephaly. A missense mutation (p.F452L) in the bone morphogenetic protein 3 (*BMP3*) gene was identified as the skull modifying locus on CFA 32. This allele is fixed in Bulldogs and French Bulldogs and has an allele frequency of 0.99 in Boston Terriers [27]. The mutations underlying the associations on CFA5, CFA24 and CFA30 have not been reported. A second group mapped proportional snout length in a GWAS across dog breeds using size as a covariate and identified both the CFA1 and CFA5 loci; however underlying causative mutations were not defined [28]. Recently, a LINE-1 insertion in the SPARC-related modular calcium binding 2 (*SMOC2)* gene was identified as the causative mutation underlying the significant associations to brachycephalic head morphology on CFA 1 [29].

In humans, a rare genetic disorder called Robinow syndrome shares phenotypic similarities with the screw tail breeds. Robinow syndrome is characterized by mesomelic-limbed dwarfism and abnormalities of the head, face, genitalia, and vertebral column [30]. Patients with Robinow syndrome have hypertelorism with a broad nasal root and broad forehead and fusion of thoracic vertebrae with frequent hemivertebrae [31]. Mutations in genes from WNT pathways, including *ROR2*, *FZD2*, *WNT5A*, *DVL1*, and *DVL3*, have all been found to cause Robinow syndrome in humans [30, 32–34]. WNT pathways control embryonic morphogenesis by regulating crucial developmental processes including cell-fate determination, proliferation, and morphogenetic cell/tissue movement [35–40].

Since the development of SNP genotyping arrays for dogs, the standard approach to mutation identification has been GWAS followed by Sanger or whole genome sequencing to identify causative variants [41]. Compared to other species, long linkage disequilibrium (LD) within the domestic dog allows successful associations with few samples and few SNPs [42]. However the long LD within dogs also presents challenges for mutation identification. In this paper, we by-pass the use of SNP genotyping arrays and utilize variant calls from whole genome paired end sequences from 100 canine samples of 21 breeds to perform whole genome variant association in the screw tail breeds. We report the identification of a frameshift mutation in the *DISHEVELLED 2 (DVL2)* gene that segregates with the breed defining phenotype of screw tail and vertebral malformations. The frameshift mutation preserves the majority of the DVL2 protein but replaces the last 49 amino acids in the C-terminus with a novel 26-amino acid sequence. Through biochemical analysis of the bulldog DVL2 variant protein in cultured cells, we further demonstrate that the mutant protein has a reduced capacity to undergo WNT-dependent phosphorylation, suggesting that aspects of WNT signaling might be compromised. Our data suggest that the bulldog-related breeds share similar genetic, morphological and pathological origins with human Robinow syndrome.

## Results

### Phenotype

**Fig 1:**
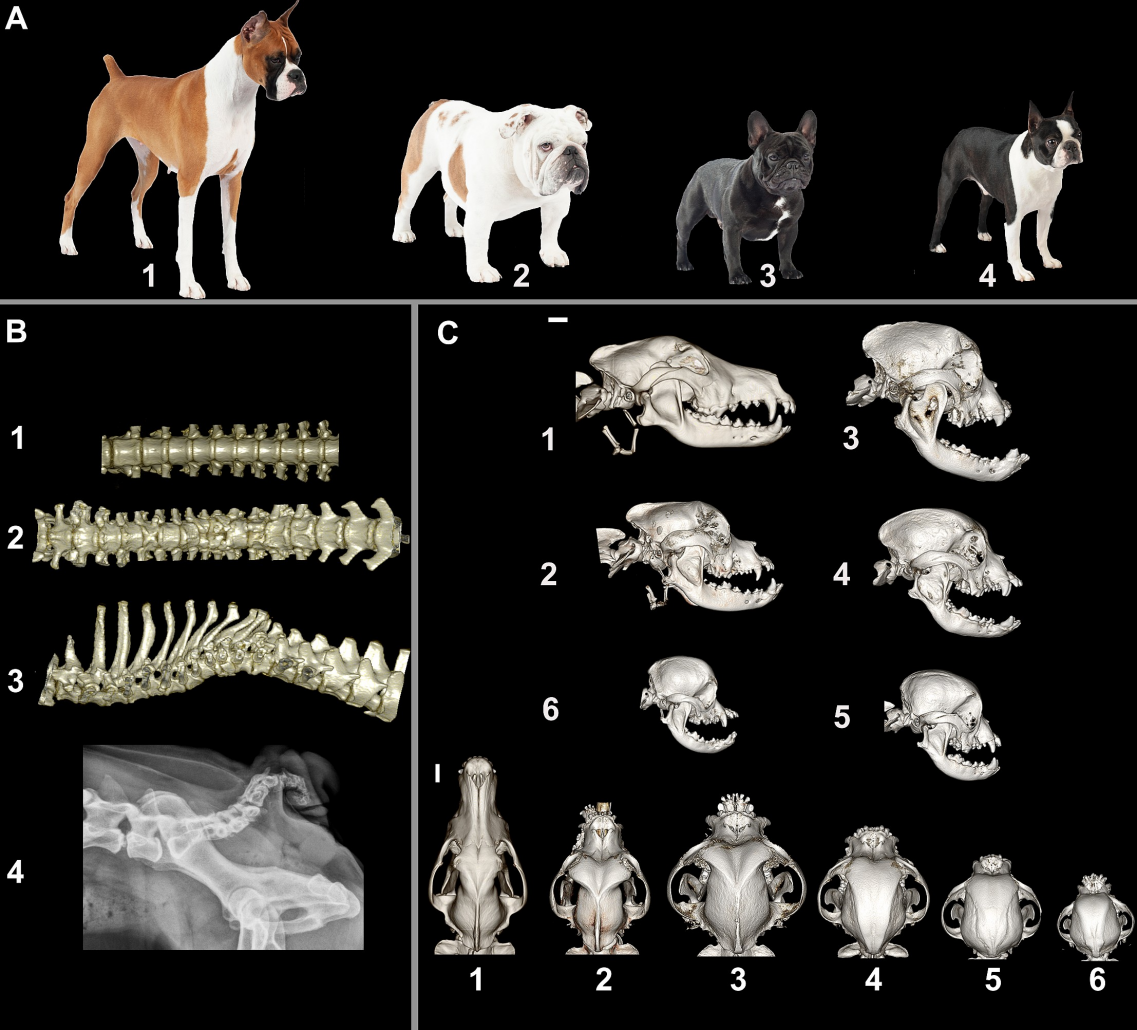
Phenotype of screw tail breeds. (A) Photographs of typical representatives of breeds: 1. Boxer (brachycephalic and normal but docked tail), 2. Bulldog (brachycephalic and screw tail), 3. French Bulldog (brachycephalic and screw tail), and 4. Boston Terrier (brachycephalic and screw tail) (photographs courtesy of Nestle Purina PetCare).(B) 3D computed tomography reconstructions of 1) Boxer thoracic vertebrae, ventrodorsal view, 2) French Bulldog thoraco-lumbar vertebrae, ventrodorsal 3) French Bulldog thoraco-lumbar vertebrae lateral view. Pronounced kyphosis of the French Bulldog vertebral column is associated with multiple vertebral abnormalities including shortened vertebrae, hemivertebrae and butterfly vertebrae. 4) Lateral vertebral column radiograph of a Bulldog demonstrating the breed typical "screw tail" associated with multiple caudal vertebral malformations and truncation. (C) Computed tomography images of canine skulls showing the variation of skull morphologies: 1) German Shepherd (dolichocephalic), 2) Boxer 3) Bulldog 4) French Bulldog 5) Boston Terrier 6) Pug. White scale bar is 2 cm for each orientation.

Bulldogs, French Bulldogs and Boston Terriers are the only American Kennel Club recognized dog breeds characterized by a screw tail (Fig 1A). The ‘screw tail’ is caused by a variety of malformed and fused vertebrae and lack of approximately 8 to 15 caudal vertebrae, which normally form the canine tail (Fig 1B 4). In addition to deformities of the caudal vertebrae, these breeds may also have variable morphological deformities of vertebrae along the vertebral column including hemivertebrae, wedge vertebrae, butterfly vertebrae and fused vertebrae (Fig 1B 1–3). Based on breed standards, the three breeds share physical characteristics including short stature, head shape and include eyes to be as wide apart as possible (hypertelorism) and for them to have a broad muzzle (Fig 1A and S1 Table). These breeds are considered brachycephalic meaning that they have a short muzzle length. The degree of brachycephaly in the three screw tail breeds is more prominent than in some other brachycephalic breeds due to an even shorter and broadened maxilla, broadened frontal bone, and an increased curvature to the zygomatic bone creating a wider and more extreme orbit as compared to, for example, the Boxer breed (Fig 1C). Due to the similarity in phenotype and historical relationships between these breeds, we hypothesized that they would share a mutation responsible for their morphology.

### Variant calling and calculation of identity by state distance

To identify variants responsible for the screw tail phenotype, we used paired end whole genome sequence data generated from 100 dogs from 21 breeds followed by association analysis of all biallelic variants identified. There were 6 trios included in the dataset. The twenty-one breeds included 5 Bulldogs, 3 French Bulldogs and two Boston Terriers. The remainder of the dogs did not have screw tails; however, two of the breeds were brachycephalic (Boxer and Pug). A complete list of breeds is available in S2 Table.

**Fig 2:**
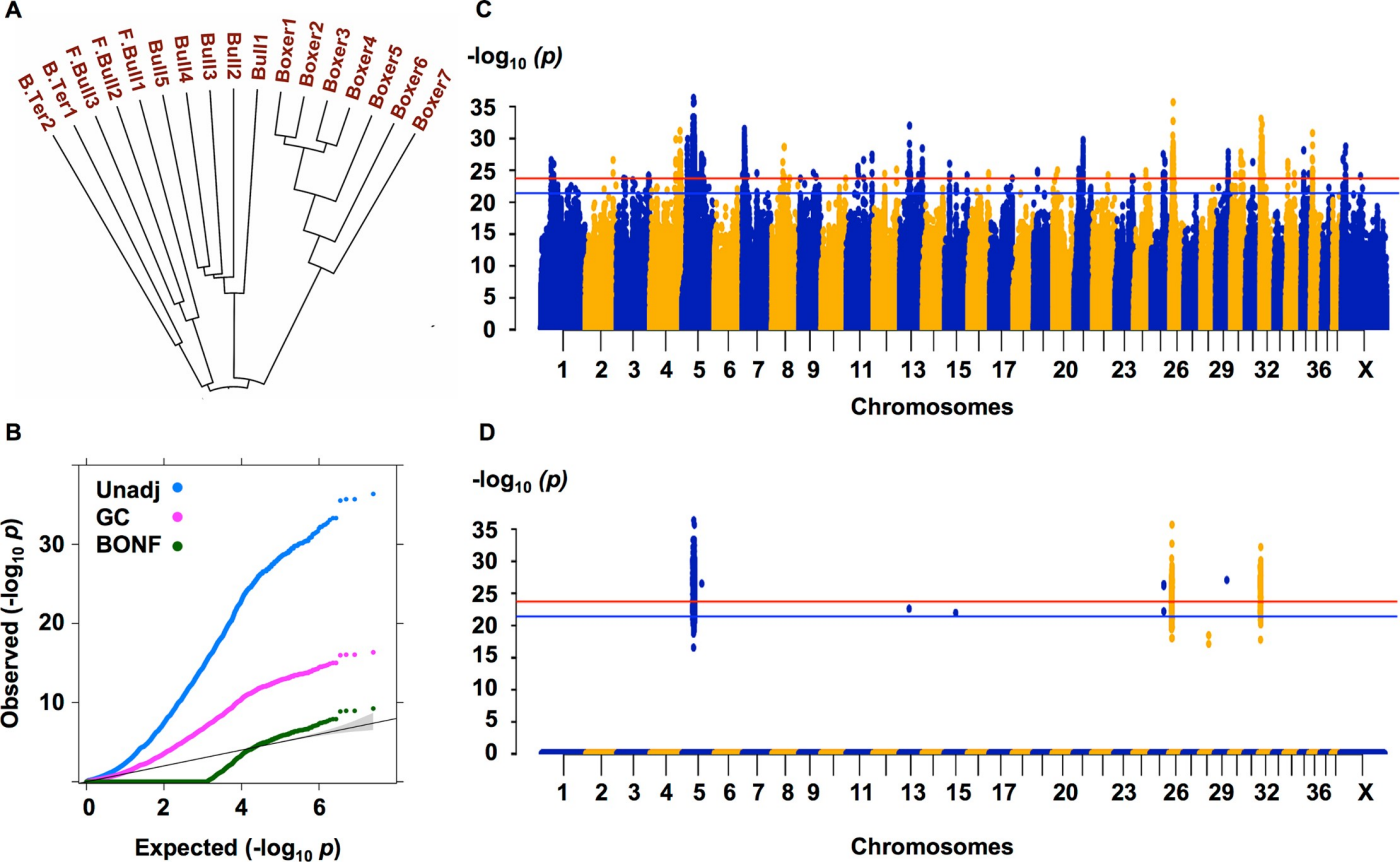
Across breed genome-wide association for screw tail. (A) Hierarchical clustering of Identity by state distance of screw tail breeds. (B) QQ plot of–log 10 of p values before (Unadj) and after genomic (GC) and statistical correction (BONF). (C) and (D) Manhattan plots with–log 10 of p values on y axis and chromosome on the x axis with horizontal blue and red lines indicating p values of 0.05 and 0.01 after correction showing all tested genotypes (C) or 90% allelic difference (D).

The variant calling pipeline identified 15,353,085 SNPs and 8,514,447 indels within the 100 canine genomes. After quality filtration and exclusion of variants of uncharacterized chromosomes, 13,591,986 SNPs and 7,126,341 indels passed our filters. The average rates of Mendelian errors per meiosis were calculated to be 4.1% in the six dog trios. A dendrogram was constructed to examine the historical relatedness of the breeds. Clustering of dogs by their breed and the relatedness in the case of the trios confirms the sensitivity of the technique to detect genetic relatedness (S1 Fig). The dendrogram highlights a common origin of all screw tail breeds (Bulldogs, French Bulldogs and Boston Terriers) and shows the bifurcation from Boxer dogs and Pugs, the other two brachycephalic breeds (Fig 2A and S1 Fig).

### Whole genome variant association

A whole genome association analysis was performed using the 10 dogs of the screw tail breeds compared to 84 dogs belonging to 21 pure and 4 mixed breeds (related dogs were removed). The association analysis started with 20,342,844 biallelic variants identified through the whole genome sequence (excluding 375,483 multi-allelic loci) with a total genotyping rate of ~93.6%. 3,023,667 variants were removed due to missing genotype data and 4,852,941 variants were removed for low minor allele frequency. Association testing was performed using the remaining 12,461,460 variants. Genomic inflation was high (estimated lambda = 3.2761) due to the expected population stratification. To minimize false positive associations caused by genomic inflation, a simple genomic control approach was used instead of other modeling approaches that correct for relatedness to avoid the exclusion of variants that were identical by descent. Since there is relatively long linkage disequilibrium in dogs, statistical thresholds were further corrected for multiple testing of 587,159 variants representing common haplotypes in the tested population. After adjustment, a P_Bonferroni_ of 0.05 is equivalent to a *p* = 3.99 X 10^−22^ and P_Bonferroni_ of 0.01 is equivalent to a *p* = 1.96 x 10^−24^ (Fig 2B, Fig 2C). A list of all the significantly associated variants is shown in S3 Table. The most associated variant was CFA5: 32195043_32195044del, *p* = 4.37 X 10^−37^ (uncorrected); however there were many associated regions (Fig 2C). This variant remained significantly associated at a P_Bonferroni_ of 0.01 corrected for all 12,461,460 variants as well (*p* = 1.5 x 10^−28^).

In addition to being highly associated, we predicted that variants responsible for breed defining characteristics would have a high allele frequency in affected breeds and an extremely low allele frequency in unaffected breeds. We therefore selected variants with more than 90% allelic difference between cases and controls. Long regions of fixed candidate variants were then selected for further analysis and included CFA5, CFA26 and CFA 32 (Fig 2D). There were two additional significant loci on CFA 25 and 29 that were not evaluated further since they were less significant and did not show long regions of homozygosity to support Identical by descent inheritance [43]. The two peaks on CFA 26 (6772912–10171935) and CFA 32 (4533724–6620950) were previously reported to be associated in these breeds with canine glioma and brachycephaly respectively [5, 27]. The highest association was to the region on CFA 5, which also had a long region of homozygosity (5:29243555–34607475). The single most significantly associated variant found was a frame shift mutation in the *Dishevelled 2* (*DVL2)* gene (g.32195043_32195044del). The mutation was homozygous in all cases and absent from controls except a single Labrador retriever that was called as heterozygous in the absence of supporting read coverage (S3 Table). This dog was later confirmed by Sanger sequencing to be wild-type at this location.

### *DVL2* mutation

**Fig 3:**
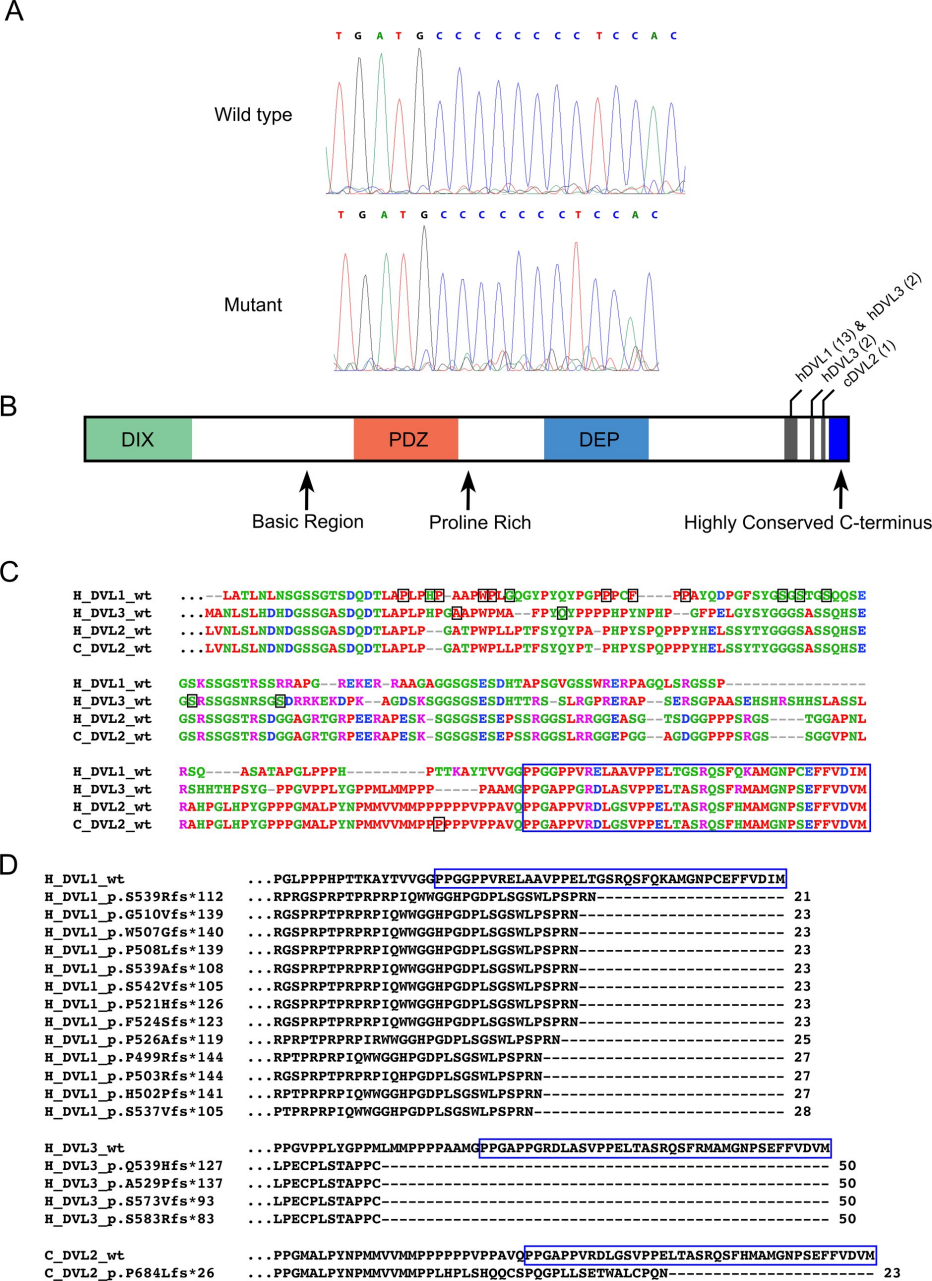
*Dishevelled 2* mutation, location within the protein and amino acid alignment with comparison to Robinow Syndrome. (A) Electropherograms of the single base pair deletion within the cDNA of *DVL2*. (B) Schematic representation of Dishevelled protein domains. Approximate locations of known mutations are marked by vertical grey bars within their respective *Dishevelled* gene (h–human; c–canine). The number of reported clinical mutations in that region, in their respective gene, is in parentheses. (C) Human wild type DVL1, DVL2, and DVL3 C-terminus protein sequences (192, 221, and 216 amino acids respectively) aligned with canine wild type DVL2 (221 amino acids). Colors of amino acids indicate their respective physiochemical property: red–small, hydrophobic; blue–acidic; magenta–basic; green–hydroxyl, sulfhydryl, amine. The location of the Dishevelled frameshift mutations (specific amino acid) are boxed in black. The highly conserved C-terminus is boxed in blue. (D) Altered amino acid sequence of human Robinow syndrome mutations in DVL1 and DVL3 and the canine DVL2 mutation identified in this work. Truncated amino acids marked by black dashes. Highly conserved region is indicated in the wild type sequence by a blue box.

The single base deletion found on CFA 5(g.32195043_32195044del) that was homozygous in the three screw tail breeds (5 Bulldogs, 3 French Bulldogs and 2 Boston Terriers) was located within the 15^th^ and penultimate exon of the canine *DVL2* gene. *DVL2* cDNA was sequenced from the skeletal muscle of a dog with a normal tail and a screw tail Bulldog (Fig 3A) to confirm the presence of the mutation in the mRNA in the Bulldog sample (*DVL2*c.2044delC). In orde to determine if there was a difference in transcript level between animals with the *DVL2*c.2044delC mutation and without semiquantitative RT PCR was performed. Mutant transcript levels were comparable to wildtype (S2 Fig). This deletion is predicted to lead to a frameshift mutation, causing a premature stop codon that truncates the translated protein by 23 amino acids (p.Pro684LeufsX26). In addition, 26 altered amino acids are predicted to be present in the highly conserved C-terminus of the mutant protein (Fig 3B, Fig 3C). The location of the truncation of the protein is remarkably similar to the effect of mutations within the other Dishevelled family members, *DVL1* and *DVL3*, that lead to Robinow syndrome in people. Alteration of the amino acid sequence in the highly conserved C-terminal region as well as truncation of 21 to 50 amino acids also occurs in the human Robinow *DVL* mutations (Fig 3D and S4 Table).

To confirm the association of the *DVL2*c.2044delC mutation with the screw tail phenotype, 667 dogs, from 49 breeds, were genotyped for the *DVL2* mutation (Table 1). 177 dogs were from the screw tail breeds including 33 Bulldogs, 79 French Bulldogs and 65 Boston Terriers. All were homozygous for the mutant allele except 6 of the Boston Terriers (4 heterozygous, 2 wildtype). In addition, we identified dogs from several other breeds, including Pit bulls, Staffordshire Bull Terrier, Shih Tzu and mixed breeds, that are heterozygous or homozygous for the *DLV2* mutation. The Pug breed has sometimes been classified with the screw tail breeds due to its curled tail; however, the tail is full length and does not have caudal vertebral malformations (S3 Fig). 29 Pugs tested were wild-type for the *DVL2* mutation. Likewise, the Pug dogs do not share the high MAF with the screw tail breeds around the *DVL2* mutation (S4 Fig). Three hundred and eighty five dogs from 43 other breeds were also tested and were all wild-type (S5 Table).

**Table 1:** Genotypes of *DVL2 (DVL2*c.2044delC) variant across breeds.

Breed	+/+	+/*dvl2*	*dvl2/dvl2*
Boston Terrier	2	4	59
French Bulldog	0	0	79
Bulldog	0	0	33
Pit Bull	18	9	6
Mixed Breed	11	2	0
Shih Tzu	27	1	0
Staffordshire Bull Terrier	0	1	0
Pug	29	0	0
43 other Breeds	385	0	0

### Segregation and penetrance

In order to determine the penetrance of vertebral malformations, dogs homozygous for the *DVL2* variant from the screw tail breeds were evaluated for thoracic and caudal vertebral malformations (Table 2). The penetrance of the thoracic malformations varied between the three breeds from 45–100%, while the caudal vertebral malformations were 100% penetrant. Since the *DVL2* variant is virtually homozygous in these breeds, it was important to evaluate segregation of the variant with phenotype. In order to confirm the association of the *DVL2* mutation with the vertebral column malformations, additional dogs were identified that had radiographs or imaging available and segregated the *DVL2* mutation. Segregation of the caudal vertebral column malformations and genotype at *DVL2* was consistent with a fully penetrant recessive mode of inheritance in Boston Terriers, Shih Tzus, Pit Bulls and mixed breeds (Table 3). Segregation of the thoracic vertebral malformations was consistent with a recessive mode of inheritance with variable penetrance between breeds. In order to evaluate the molecular contribution to brachycephalic skull shape, the *DVL2* mutant breeds were genotyped for the previously identified brachycephaly associated Line insertion affecting SMOC 2 splicing. Bulldogs and French Bulldogs were homozygous for the variant and the Boston Terriers had an allele frequency of 90.3% (S6 Table). Similar allele frequency for the *BMP3* missense mutation was also reported for these three breeds indicating that they are homozygous or have a high allele frequency for three mutations that affect head shape [27].

**Table 2:** Penetrance of Vertebral Malformations within dogs homozygous for DVL2c.2044delC.

	Thoracic Vertebral Malformation			Caudal Vertebral Malformation		
Breed	Present	Absent	Penetrance	Present	Absent	Penetrance
Boston Terrier	20	24	45.5	12	0	100
French Bulldog	47	4	92.1	42	0	100
Bulldog	7	0	100	3	0	100

**Table 3:** Segregation Analysis of the *DVL2* (*DVL2*c.2044delC) Variant with Vertebral Phenotype.

		Thoracic Vertebral Malformation		Caudal Vertebral Malformation	
Breed	Genotype	Yes	No	Yes	No
Boston Terrier	+/+	0	2	0	1
	*dvl2*/+	0	2	0	2
	*dvl2*/ *dvl2*	20	24	12	0
Shih Tzu	+/+	0	17	0	8
	*dvl2*/+	0	1	0	1
Pit Bull and Mixes	+/+	0	14	0	7
	*dvl2*/+	0	5	0	3
	*dvl2*/ *dvl2*	0	2	4	0
Fisher’s exact test		*p* = 1.5 X 10^−5^		*p* = 4.5 X 10^−11^	

### WNT-dependent phosphorylation of the DVL2 mutant protein is reduced

**Fig 4:**
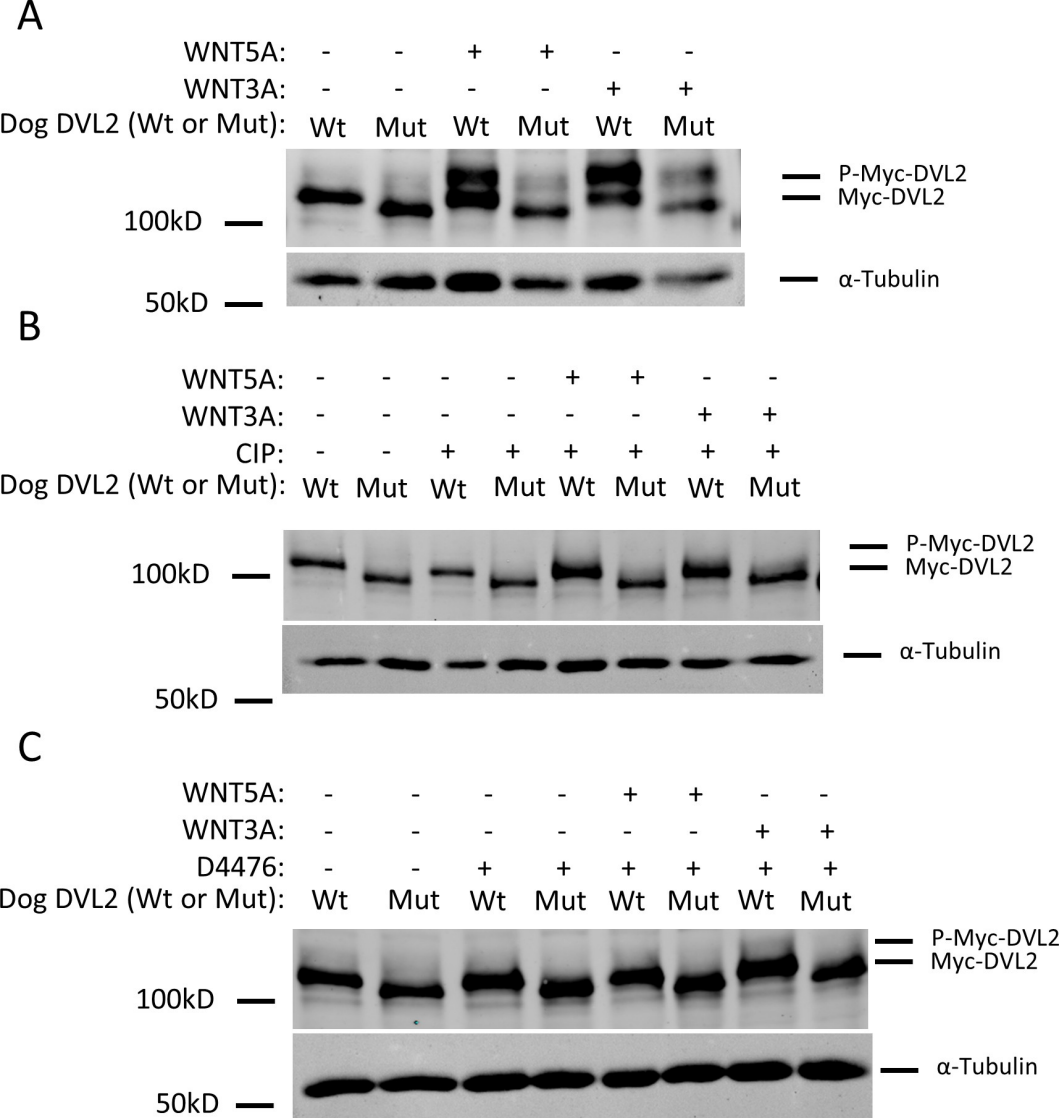
WNT-dependent phosphorylation of the DVL2 mutant protein is reduced. (A) Lysates from NIH/3T3 stable cell lines expressing the dog, Myc-tagged wild-type (Wt) or mutant variant (Mut) DVL2, which is 23 aa shorter than wild-type exogenous DVL2, were analyzed by western blotting using an anti-c-Myc antibody. To assess the ability of the wild-type and mutant proteins to respond to WNT stimulation, cells were treated with WNT5A or WNT3A for 6 hours. Both treatments resulted in increased gel mobility shifts of the wild-type DVL2 protein, indicative of increased phosphorylation; this effect was reduced on the mutant DVL2 protein. (B) To confirm that the DVL2 gel mobility shifts observed in (A) were due to phosphorylation, cell lysates were subjected to mock treatment (30 min incubation at 37 C), or calf intestinal phosphatase (CIP) treatment (30 min incubation at 37 C in the presence of CIP) before separation by SDS-PAGE. The DVL2 gel mobility shifts above wild-type and mutant proteins were lost after CIP treatment, confirming that they are caused by phosphorylation. (C) To test whether the DVL2 gel mobility shifts observed in (A) were driven by casein kinase 1 (CK1), cells were treated with D4476, a CK1 inhibitor, for 1 hour prior to and concurrently during the Wnt stimulation for 6 hours. The DVL2 gel mobility shifts were lost after D4476 treatment, further indicating that they are caused by CK1-dependent phosphorylation. α-tubulin was used for loading controls. Cell lysates were normalized by BCA assays for total protein.

Based on the significant changes to the DVL2 C-terminus caused by the frameshift mutation, we hypothesized that the expression and/or biochemical properties of the mutant variant DVL2 might be altered. To test this hypothesis, we synthesized full-length cDNA of both the wild-type and mutant variant dog *DVL2* gene, N-terminally tagged them with the MYC epitope, and expressed them via lentiviral vectors in NIH/3T3 cells. We chose to use NIH/3T3 cells because they have been used previously to study WNT pathways and DVL regulation [44, 45]. Western analysis of lysates from cell lines expressing a wild-type or mutant variant of DVL2 showed that both proteins were expressed at similar levels (Fig 4A), suggesting that the mutant protein is properly synthesized and its novel C-terminus does not affect the stability of the DVL2 protein.

It has been previously established that Wnt stimulation results in the phosphorylation of DVL2, which can be observed on western blots as gel motility shifts and is often used as an indicator of pathway activity [46–49]. To compare the capacity of wild-type and mutant variant DVL2 to respond to WNT signals, we stimulated cells expressing these proteins with purified, recombinant WNT5A or WNT3A and analyzed the extent of DVL2 gel mobility shifts on western blots. We observed prominent DVL2 gel motility shifts in cells expressing the wild-type DVL2 protein after WNT5A or WNT3A treatment (Fig 4A). However, DVL2 gel motility shifts were reduced in cells expressing the mutant protein, suggesting that phosphorylation of the mutant variant DVL2 may be impaired. To demonstrate that the gel motility shifts observed were indeed due to phosphorylation, we treated cell lysates with calf intestinal phosphatase (CIP) to remove any potential phosphate groups. CIP treatment resulted in loss of the slower migrating DVL2 bands and an increased amount of unphosphorylated DVL2 in all conditions (Fig 4B). These results indicate that Wnt-dependent phosphorylation of the mutant variant DVL2 protein is reduced compared to wild-type DVL2.

Additional studies conducted in human and mouse cells have demonstrated that WNT-dependent phosphorylation of DVL2 is dependent on casein kinase 1 (CK1) [50, 51]. To assess if this mechanism also drives phosphorylation of the canine DVL2 protein, we pretreated cells with D4476, a small molecule inhibitor of CK1, and then stimulated with either WNT5A or WNT3A. D4476 treatment resulted in the loss DVL2 phosphorylation in all conditions, thereby demonstrating that CK1 also mediates Wnt-dependent phosphorylation of the canine DVL2 protein (Fig 4C). Collectively, these results demonstrate that the canine mutant variant DVL2 protein exhibits reduced WNT- and CK1-dependent phosphorylation and further suggest that reduced WNT signaling may contribute to the Robinow-like phenotype of bulldogs and associated screw tail breeds.

## Discussion

Whole genome sequence data from 100 dogs was utilized to interrogate the molecular cause of the breed defining trait screw tail. Using over 12 million bi-allelelic variants and comparing 10 cases to 84 controls, a frame shift mutation in the *DVL2* gene was found to be the most strongly associated of all variants. The mutation leads to a 23 amino acid truncation of the protein in the last exon within the highly conserved C-terminal domain. Analogous truncations in the same regions of human DVL1 and DVL3 proteins result in Robinow syndrome in humans. This shared genetic signature, taken together with the similar anatomical changes, strongly suggests that the bulldog-related breeds share common pathological origins with human Robinow syndrome.

DVL2 is part of an evolutionarily conserved cytoplasmic scaffolding protein family that also includes DVL1 and DVL3 in vertebrates [52, 53]. The three mammalian DVL homologs display significant sequence identity to each other [54, 55] as well as across species [56, 57]. Our results suggest that the mutant variant DVL2 protein likely has reduced capabilities to mediate WNT signaling. Although DVL proteins are key players in a variety of WNT pathways, the Robinow-like phenotypes of bulldogs and human patients coupled with mouse developmental expression patterns lead us to believe that the frameshift mutations in DVL2 in screw tail dog breeds primarily affect a noncanonical branch of WNT signaling, the WNT5A-ROR pathway. In addition to the bulldog DVL2 frameshift mutation, human mutations causing Robinow syndrome have been identified in *WNT5A*, *ROR2*, *FZD2*, *DVL1*, and *DVL3*, which are all major components of the WNT5A-ROR pathway [32, 33, 58–61]. Further, WNT5A and ROR2 are highly expressed in the facial primordia, limb buds, and vertebrae of mice, all areas of the body that are affected in Robinow syndrome patients and bulldogs; additionally, *Wnt5a* and *Ror2* knockout mice exhibit Robinow-like features, including truncated limbs and broad, flat faces [44, 46]. Collectively, this suggests that screw tail dog breeds and Robinow syndrome patients at least partly share similar underlying pathophysiology.

In both bulldogs and human patients, frameshift mutations in the penultimate or ultimate exon of DVL proteins result in the substitution of the highly conserved C-terminus region of the proteins with a new stretch of amino acids. Given that the frameshift mutation leaves most of the protein intact, it remains plausible that the more N-terminal modular domains of DVL2 can still function in other Wnt pathways, such as canonical WNT/beta-catenin signaling, which primarily uses the DIX domain, and planar cell polarity, which primarily uses the DEP domain [53]. Beyond these modular domains, however, few roles have been assigned to the DVL2 C-terminus; work by Bernatik et al has shown that human DVL3 C-terminus contains some CK1 phosphorylation sites that are conserved in DVL2 and would be lost by the frameshift mutation [62]. This correlates with our observations that the mutant DVL2 variant exhibits reduced CK1-dependent phosphorylation in response to WNT stimulation and is consistent with a recessive mode of inheritance. Given the importance of DVL2 in WNT5A-ROR signaling and the potential roles that these pathways play in defining the unique phenotypical and pathological characteristics of the screw tail bulldog breeds, additional follow up studies are required to define the molecular mechanism(s) by which the DVL2 variant protein affects downstream WNT signaling activity during development.

In addition to the *Dvl2* frameshift mutation, the three Bulldog related breeds harbor other mutations in developmentally important genes that could affect their craniofacial morphology. For example, these breeds also carry a missense mutation in *BMP3* and a Line 1 insertion that affects splicing of the *SMOC2* gene [27, 29]. The absence of homozygosity for these mutant alleles in breeds fixed for the brachycephalic head phenotype indicates that this is a complex trait influenced by multiple loci. Screw tail breeds are distinguished from some of the other brachycephalic dogs by additional shortening and broadening of the muzzle, broadening of the skull, and hypertelorism suggesting the presence of more variants affecting their skull morphology (Fig 1A, S1 Table and [27, 63]). We did not undertake the evaluation of head morphology in this work since recruiting pet dogs for head CT that is not medically warranted is ethically challenging since it requires general anesthesia and carries a risk to dogs with brachycephalic head conformation. However, previous across breed genome wide associations for head morphology identified this locus on CFA 5 [27, 28]. Boyko et al used breed-based averages for a large number of linear measurements followed by QTL mapping [28]. Schoenebeck et al performed principal component analysis of geometric morphometry of museum specimen skulls from breeds followed by GWAS using DNA samples from the same breeds [27]. Both groups identified the same highly associated SNP (CFA5:32359028) within 160 Kb of the *DVL2* mutation as having a significant contribution to skull shape in dogs. A third study on canine skull morphology that was based on individual measurements taken from skull CTs did not identify the CFA 5 locus; however, the screw tail breeds only made up ~2% of the sample sets used in this across breed study to identify QTLs that affect head morphology [29]. Since the study was designed to capture common loci across many breeds it is not surprising that the *DVL2* locus would not be identified. Based on our allele frequency measures, the mutation is rare across breeds and virtually homozygous in affected breeds.

Additional, as yet undiscovered, deleterious variants could be present in these breeds that affect their skull shape considering the extremely strong selection based on head phenotype applied to these breeds by dog breeders. Normal development of the skull requires coordinated development of cranial sutures, skull base synchondroses and brain, and it is likely that genetic abnormalities may affect both suture and synchondrosis development directly, or indirectly due to secondary effects [64, 65]. WNT signaling has been shown to regulate cranial base development and growth, and abnormalities in WNT signaling have been implicated in cranial synostosis in humans, most commonly with brachycephaly-associated coronal synostosis [66–68]. Given the apparent polygenic pathogenesis of brachycephaly in dog breeds, and the essentially fixed nature of the *DVL2* mutant allele in the Bulldog and Boston breeds, assigning specific skull morphometric sequelae to the *DVL2* mutation is challenging. The cranial dysmorphology seen in Robinow patients and the more extreme nature of the brachycephaly in the *DVL* mutant dog breeds is, however, highly suggestive of *DVL2*’s involvement in the brachycephalic phenotype. A more detailed study of skull morphology, particularly in animals segregating the various associated genes isnecessary to define specific gene contributions to the brachycephalic phenotype in dogs.

Although caudal vertebral malformations appear to be a consistent finding within *DVL2* mutant dog breeds, thoracic vertebral malformations are more variable in their presence and severity. Similar findings are observed in mouse models and human patients with WNT pathway abnormalities where penetrance of vertebral anomalies is variable. Even in highly inbred *Dvl2* knockout mice, only 90% were reported to have vertebral anomalies [69], and hemivertebrae and associated scoliosis/kyphosis are seen variably in >75% and <25% of cases with recessive and dominant forms of Robinow syndrome respectively [70]. In addition to vertebral abnormalities, Dvl2 knockout mice exhibit 50% perinatal lethality due to cardiac defects, 25% have tail kinks and 4% have tail truncations [69]. Based on the presence of protein product found in MYC tagged experiments, we propose that the canine DVL2 mutation is not a null mutation but rather a hypomorphic mutation with respect to vertebral malformations. This is also consistent with the more severe phenotype seen in *DVL2* knockout mice. The possibility exists that there are different effects in different developmental pathways or tissues, and possibly, polygenic effects due to the presence of many segregating mutations that are already known to affect size and skull shape in domestic dogs.

The ability to perform whole genome association in dogs allowed the elimination of the fine structure mapping step in causative variant identification. This whole genome variant association approach successfully replicated two previously identified loci that were known to be fixed within the screw tails breeds namely the *BMP3* missense mutation (CFA32) associated with head morphology and the glioma susceptibility locus (CFA26) [5, 27]. This approach is particularly tractable in the dog where deleterious variants are shared within and across breeds and only a single causative variant is expected. However, it should be noted that only SNPs and small insertion deletions were identified in this analysis, and there are many examples of disease causing variants that would not have been identified using this approach [6, 29]. As whole genome sequencing costs continue to decrease and our abilities to call variants improves, whole genome variant association provides an efficient method to define disease causing variants.

## Materials and methods

### Ethics statement

The following application was reviewed and approved by the UC Davis IACUC on January 19, 2018. Title: Canine DNA collection from privately owned animals. Principal Investigator: Danika L. Bannasch (Protocol # 20356) Institution: University of California, Davis. Active protocols are reviewed annually. This institution is accredited by the Association for Assessment and Accreditation of Laboratory Animal Care, International (AAALAC). This institution has an Animal Welfare Assurance on file with the Office of Laboratory Animal Welfare (OLAW). The Assurance Number is A3433-01. The IACUC is constituted in accordance with U.S. Public Health Service (PHS) Animal Welfare Policy and includes a member of the public and a non-scientist.

### Canine samples

Buccal swabs or blood samples were collected from privately owned dogs through the William R. Pritchard Veterinary Medical Teaching Hospital at UC Davis. Owners specified the breed of each dog. Samples used for whole genome sequencing included 96 dogs from 21 pure breeds and 4 dogs from mixed breeds (S2 Table).

### Phenotype

Images of screw tail breeds were obtained during the course of necessary veterinary procedures and 3D computed tomography reconstructions were perfomed on selected images. The American Kennel Club breed standards were used as a guide to describe typical dogs of each breed. Vertebral column phenotypes defined as a) thoracic vertebral malformation and b) caudal malformation (“screw tail”) were based on assessment of imaging (radiographs, computed tomography or magnetic resonance imaging) by a board certified veterinary radiologist and a board certified veterinary neurologist. Cases were considered affected if there was evidence of thoracic vertebral malformations characterized by the presence of wedged vertebrae, hemi-vertebrae or butterfly vertebrae, or the presence of similar malformations as well as shortening and vertebral fusions affecting the caudal vertebrae. Inclusion required the availability of imaging for all thoracic vertebrae in lateral and dorso-ventral /ventro-dorsal planes for thoracic vertebrae, and imaging of a minimum of 6 caudal vertebrae. Phenotyping of 4 cases for the presence of caudal malformation (“screw tail”) was based on visual and physical examination by a veterinarian.

### DNA extraction and whole genome sequencing

Genomic DNA was extracted using the Qiagen kit (QIAGEN, Valencia, CA). 96 biological samples (including 6 trios) were subjected to next generation sequencing using Illumina paired end cycles. The whole genome sequencing of 4 Pug samples was publically available by Tgen company (https://www.tgen.org/). The metadata table contains details about the sequencing libraries and coverage (S2 Table).

### Variant calling

Adaptors and low quality sequences were removed using the Trimmomatic software (V 0.36)[71]. Adaptor trimming was done using recommended parameters of simple matching (threshold of 10) and palindromic matching (threshold of 30 and minimum adapter length of 1). High quality reads were aligned to the dog reference genome CanFam3 [72] using the BWA-MEM algorithm of the BWA software package (v0.7.7) [73]. Duplicate reads were excluded using the Picard tool MarkDuplicates (v2.2.4) (http://broadinstitute.github.io/picard). Variant calling was performed with the GATK HaplotypeCaller (v3.5) [74] using joint genotyping across all sequenced samples. Known variants from the Ensembl variation database (release 82) [75] and canine annotation of Broad institute (https://www.broadinstitute.org/ftp/pub/vgb/dog/trackHub/canFam3/variation/final.Broad.SNPs.vcf.gz) were used for variant annotation. Candidate variants were filtered using the following thresholds: QualByDepth (QD) < 2.0, FisherStrand (FS) > 60.0, StrandOddsRatio (SOR) > 4.0, ReadPosRankSum < -8.0, and depth of coverage (DP) > 3105 for both SNPs and indels, RMSMappingQuality (MQ) < 40.0, MQRankSum < -12.5 for SNPs, and InbreedingCoeff < -0.8 for indels. After quality filtration and exclusion of variants of uncharacterized chromosomes, 13,591,986 SNPs and 7,126,341 indels passed our filters.

### Including indels in the PLINK analysis

The PLINK software for GWAS is designed to deal with SNPs for association studies[76]. To allow PLINK to deal with indels as well, we developed a script which changed the bi-allelic indels into SNPs. Here, we excluded multi-allelic variants then replaced multi-character alleles by a single character (A or T) chosen to maintain allelic variation between reference and alternatives (https://github.com/dib-lab/dogSeq).

### Identity by state (IBS) distance

An IBS matrix was calculated to document the breed-based population stratification and examine the similarities between the breeds used. A subset of autosomal and X chromosome variants genotyped in >95% of samples with MAF > 5% was selected and then subjected to linkage disequilibrium based pruning using a threshold of variance inflation factor (VIF) equals 2. Pruning recursively removed SNPs within a sliding window of 50 SNPs, with a window step size of 5 SNPs producing 645,697 variants for IBS calculation. The distance matrix was constructed using the ‘—distance 1-ibs’ function of PLINK 1.9 and plotted as a dendrogram using the ‘ape’ package in R. The “—mendel” option in PLINK 1.9 was used to calculate the rate of Mendelian errors per meiosis in the sequenced trios using the pruned subset of variants. The average rate of Mendel errors was used as an index for the genotyping accuracy.

### Genome wide association using variants identified by whole genome sequence

Among the 100 dogs sequenced, there were 6 trios whose offspring were excluded from further analysis. All variants were subjected to mild filtration to exclude those failing to genotype in more than 10% of all sequenced samples as well as those with MAF of less than 1%. Following this, we used PLINKv1.9 to perform a case/control association analysis (S3 Table). Statistical probabilities were adjusted for genomic inflation using the Genomic Control (GC) approach GC correction is based on the assumption that most of variants are not associated with the trait of interest and thus the chi-square values of statistical tests should have a mean of one. Genomic inflation increases this mean and to correct for this, all test statistics values are divided by the mean of the test statistics to restore the expected distribution [77]. Bonferroni correction for multiple testing was performed using pruned variants as an index for independently tested haplotypes to obtain a list of candidate loci [78]. Pruning was done as described above for IBS calculations but after excluding the offspring of the 6 trios. All variants belonging to the same haplotype are dependent and should have similar association probabilities. Correction of multiple testing should be applied to the “independent” statistical trials. In our experiment, correcting for all tested variants did not prevent the detection of the causative variant (*p* = 4.37 X 10^−37^ uncorrected) even with a threshold of 0.01 after Bonferroni correction. Fixed variants in affected breeds that were approaching absence from unaffected breeds were selected as those with more than 90% allelic differences between cases and controls.

### Variant effect annotation

The Variant Effect Predictor (VEPv85) tool from Ensembl [79] was used to annotate possible effects of all detected variants. Variant annotation was done using the NCBI dog genome annotation (last modified on 9/18/15)[80].

### Genotyping

Primers were designed using Primer3 [81]. Primers to amplify the *DVL2* mutation produced a 297 base pair product (Forward Primer: CGGCTAGCTGTCAGTTCTGG; Reverse Primer: CAGTGAGTCTGAGCCCTCCA). PCR products were sequenced using the Big Dye termination kit on an ABI 3100 Genetic Analyzer (Applied Biosystems, Foster City, CA). Segregation analysis was evaluated by Fisher’s exact test. Sequences were evaluated using Chromas (Technelysium, South Brisbane, QLD, Australia). The sequences were aligned to the Boxer dog reference sequence (CanFam 3.1) using BLAT (UCSC Genome Browser). Primers described by Marchant et al. [29] were used to evaluate the SMOC2 mutation status for 152 dogs. PCR products sizes were visualized via gel electrophoresis.

### RNA extraction and cDNA Sequencing

All primers were designed using Primer 3 (Forward Primer: CCACGAGCTGTCATCCTACA; Reverse Primer: CAACTGACAGGGCAGACAGA) [81]. RNA was isolated from skeletal muscle and spleen using Qiagen QIAamp Blood Mini Kit tissue protocols (QIAGEN, Valencia, CA). RNA was reverse transcribed into cDNA using Qiagen QuantiTect Reverse Transcription Kit. *DVL2* and *RPS5* [82] cDNA was PCR amplified from skeletal muscle tissue from one Bulldog and one Labrador Retriever. *RPS5* was amplified in skeletal muscle to ensure equivalent amounts of cDNA were produced. The PCR products were sequenced on an ABI 3500 Genetic Analyzer and analyzed using Chromas (Technelysium, South Brisbane, QLD, Australia). The sequences were aligned to the reference Boxer dog genome (Can Fam 3.1), using BLAT (UCSC Genome Browser), to confirm sequences matched *DVL2*. Semiquantitative RT PCR was performed by PCR amplification (Forward Primer: CGAGCTGTCATCCTACACCT, Reverse primer: TGACGAGCCTCTGGAAGG) of cDNA from Spleen from two different dogs of each genotype (homozygous mutant and wildtype for *DVL2*c.2044delC). Reduced PCR cycle number (to 28) were used to estimate the transcript differences. PCR products were visualized on agarose gels.

### *DVL2* gene cloning

Due to extremely high GC-content, attempts to clone the canine *DVL2* open reading frame (ORF) were unsuccessful. We therefore commercially synthesized the wild type and the bulldog variant of *DVL2* and subcloned them into a modified pENTR-2B vector containing an N-terminal Myc tag (MT) using the FseI and AscI restriction sites. The ORFs were verified by Sanger sequencing. To generate lentiviral transfer vectors, the pENTR-2B-MT constructs were recombined with the pLEX_307 vector (a gift from David Root; Addgene plasmid #41392) using LR clonase (Thermo Fisher Scientific, Hanover Park, IL). Transgene expression from pLEX_307 is driven by the *EF1* promoter.

### Generation of stable NIH/3T3 cell lines

Lentiviruses were generated in HEK293T cells via co-transfection of lentiviral vectors with the following third generation packaging plasmids: pMD2.G (Addgene plasmid # 12259), pRSV-rev (Addgene plasmid #: 12253) and pMDLg/pRRE (Addgene plasmid #: 12251) [83]. 0.75mL of viral supernatant was used to infect NIH/3T3 cells plated at 20% confluency in 24-well plates. Puromycin selection (0.002mg/mL) was carried out for 4 days.

### Western blotting

To block synthesis and production of endogenous Wnt proteins, the porcupine inhibitor Wnt-C59 (100nM final concentration) was added to cells 24 hours prior to lysis. For WNT stimulation, cells were incubated for 6 hours with Wnt5a (R&D, catalog #645WN010, 200ng/mL final concentration) or Wnt3a (R&D, catalog #1324-WN-002 100ng/mL final concentration). For casein kinase 1 inhibitor treatment, cells were pre-treated with D4476 (APEXBIO catalog #A3342, 100nM final concentration) for 1h prior to WNT5A or WNT3A treatment. D4476 was maintained in the culture during the 6-hr WNT stimulation period. Cells were washed in 1X cold PBS and lysed in 200μL RIPA buffer supplemented with Halt™ Protease Inhibitor Cocktail (100X) (Prod # 186127, Thermo Fisher Scientific). BCA analyses were conducted to determine the absolute concentrations of protein in lysate samples. For phosphatase treatment, 17.3μg of protein from cell lysates were treated with 7 U of CIP (NEB, catalog #M0290S; final concentration of 350U/mL) at 37C for 30 minutes. Lysates subjected to mock treatment were incubated at the same temperature and duration without enzyme. Protein concentrations were normalized and lysates were mixed with 1/3 the volume of 4X LDS sample buffer (NP0008, Thermo Fisher Scientific) supplemented with 2-mercaptoethanol (4.25% final concentration). Lysates were heated at 95°C for 5 minutes before SDS-PAGE and western blots were generated.

For detecting exogenous DVL2, a commercially purchased monoclonal anti-c-Myc antibody (clone 9E10, Thermo Fisher Scientific, catalog #9801) was used as the primary antibody at a dilution ratio of 1/1000, and a goat anti-rabbit IgG polyclonal antibody (conjugated to IRDye 800CW; catalog # 926–32211, Li-cor Biosciences, Lincoln, NE) was used as the secondary antibody at a dilution ratio of 1/30,000. For detecting α-tubulin, the DM1A mouse monoclonal antibody (catalog # SC-32292, Santa Cruz Biotechnology, Dallas, TX) was used as the primary antibody at a dilution ratio of 1/1000, and a goat anti-mouse IgG polyclonal antibody (conjugated to the IRDye 800CW; catalog # 926–32210, Li-cor Biosciences) was used at a dilution ratio of 1/30,000). Imaging of the western blots was performed using the Odyssey infrared imaging system (Li-cor Biosciences) according to the manufacturer’s instructions. Non-saturated protein bands were quantified using Odyssey software, with a gamma level of 1.

## Supporting information

S1 Table Physical Characteristics of Screw Tail Breeds. (DOCX)

S2 Table Dog breeds, sequencing libraries and coverage. (XLSX)

S3 Table All significantly associated variants from GWAS. (XLSX)

S4 Table Clinical Disheveled Gene Mutations. (DOCX)

S5 Table Other breeds from Table 1. (XLSX)

S6 Table SMOC2 Line 1 insertion (CFA1:58,989,568) Genotyping. (DOCX)

S1 Fig Hierarchical clustering of Identity by state distance for all sequenced dogs. (DOCX)

S2 Fig Semi-quantitative RT PCR. (TIF)

S3 Fig Pug tail radiograph. (TIF)

S4 Fig MAF of the candidate haplotype variants for the screw tail breeds and the Pug. (TIFF)
