# Supplementary material for: Whole genome variant association across 100 dogs identifies a frame shift mutation in DISHEVELLED 2 which contributes to Robinow-like syndrome in Bulldogs and related screw tail dog breeds
Source: PLoS Genet. 2018 Dec 6;14(12):e1007850. doi: 10.1371/journal.pgen.1007850 (PMC6303079; doi:10.1371/journal.pgen.1007850)
Supplement: S1 Table — (DOCX) [file pgen.1007850.s001.docx]

**S1 Table Physical Characteristics of Screw Tail Breeds**

|  | Boston Terrier | Bulldog | French Bulldog |
| --- | --- | --- | --- |
| Weight | 15-25 pounds | 40-50 pounds | <28 pounds |
| Height | 15-17 inches | 12-16 inches | 11-13 inches |
| Tail | Short, fine, tapering, straight or screw | Straight or screwed, short, thick root and fine tip | Straight or screwed, short, thick root and fine tip |
| Eyes | The eyes are wide apart | (eyes)…”as wide apart as possible” | (eyes)…wide apart” |
| Skull Shape | Square, flat on top, flat cheeks. brow abrupt and the stop well defined. The muzzle is short, square, wide and deep and in proportion to the skull. | Very large, flat forehead, broad and square, extremely short broad muzzle. | Top of the skull is flat, slightly rounded forehead, broad muzzle, underjaw is deep, square, broad, and undershot. |
| Cranial Index^*^ | 105.2 | 107.3 | 102.1 |

^✚^(http://www.akc.org, www.purina.com/dogs/dog-breeds)

*Calculated using Stockard’s measurements and the classical cranial index calculation: (Skull Width×100)÷Skull Length (Stockard 1941, Mador, Goncim et al. 2010, Likus, Bajor et al. 2014).

∞A Cranial Index above 81 is considered brachycephalic (Likus, Bajor et al. 2014)
