# Supplementary material for: Whole genome variant association across 100 dogs identifies a frame shift mutation in DISHEVELLED 2 which contributes to Robinow-like syndrome in Bulldogs and related screw tail dog breeds
Source: PLoS Genet. 2018 Dec 6;14(12):e1007850. doi: 10.1371/journal.pgen.1007850 (PMC6303079; doi:10.1371/journal.pgen.1007850)
Supplement: S4 Table — (DOCX) [file pgen.1007850.s004.docx]

**S4 Table Clinical Disheveled Gene Mutations**

| Clinical Disheveled Gene Mutations | | | | | | |
| --- | --- | --- | --- | --- | --- | --- |
| Author | Gene | Location | Mutation | Domain | Protein Effect | Phenotype |
| (Bunn, Daniel et al. 2015) | *DVL1* | Exon 14 | c.1519del (p.Trp507Glyfs*140)  c.1562del (p.Pro521Hisfs*126) | C-terminus | Truncation of 23 AA of C-terminus | Dominant Osteosclerotic Robinow Syndrome |
|  |  |  | c.1576_1583delinsG (p.Pro526Alafs*119) |  | Truncation of 25 AA of C-terminus |  |
| (DeMarco, Merello et al. 2013) | *DVL2* | Exon 1 | c.158C>T (p.Ala53Val) | DIX | Altered 1 AA in DIX Domain | Neural Tube Defects |
|  |  | Exon 10 | c.1073C>T (p.Ser358Phe) | Proline-rich region | Altered 1 AA in Proline-rich region |  |
|  |  | Exon 15 | c.1801_1802insG (p.Glu620X) | C-terminus | Truncation of 116 AA of C-terminus | Neural Tube Defects |
|  |  |  | c.2000A>G (p.Tyr667Cys) |  | Altered 1 AA in C-terminus |  |
|  | *DVL3* | Exon 5 | c.523A>G (p.Ser175Gly) | Polypeptide Linker | Potentially Damaging |  |
| (White, Mazzeu et al. 2015) | *DVL1* | Exon 14 | c.1505_1517del (p.His502Profs*141)  c.1508del (p.Pro503Argfs*144) | C-terminus | Truncation of 27 AA of C-terminus | Dominant Robinow Syndrome |
|  |  |  | c.1519del (p.Trp507Glyfs*140)  c.1529del (p.Gly510Valfs*137)  c.1570_1571delins (p.Phe524Serfs*123)  c.1615del (p.Ser539Alafs*108) |  | Truncation of 23 AA of C-terminus |  |
| (White, Mazzeu et al. 2016) | *DVL1* | Exon 14 | c.1522delC (p.Pro508Leufs*139) | C-terminus | Truncation of 23 AA of C-terminus | Dominant Robinow Syndrome |
|  | *DVL3* | Exon 14 | c.1585delG (p.Ala529Profs*137) |  | Truncation of 50 AA of C-terminus |  |
|  |  | Exon 15 | c.1716delC (p.Ser573Valfs*93)  c.1749delC (p.Ser583Argfs*83) |  |  |  |
|  |  |  | c.1715-1G>A  c.1715—2A>G |  | Splice-altering; activation of a cryptic splice acceptor site |  |
| (White, Mazzeu et al. 2018) | *DVL1* | Exon 14 | c.1612_1616dup (p.Ser539Argfs*110) | C-terminus | Truncation of 21 AA of C-terminus | Dominant Robinow Syndrome |
|  |  |  | c.1623del (p.Ser542Valfs*105) |  | Truncation of 23 AA of C-terminus |  |
|  |  |  | c.1496_1508del (p.Pro499Argfs*144)  c.1505_1517del (p.His502Profs*141) |  | Truncation of 27 AA of C-terminus |  |
|  |  |  | c.1608_1623del (p.Ser537Valfs*105) |  | Truncation of 28 AA of C-terminus |  |
|  | *DVL3* | Exon 14 | c.1617del (p.Gln539Hisfs*127) |  | Truncation of 50 AA of C-terminus |  |
