# Supplementary material for: Whole genome variant association across 100 dogs identifies a frame shift mutation in DISHEVELLED 2 which contributes to Robinow-like syndrome in Bulldogs and related screw tail dog breeds
Source: PLoS Genet. 2018 Dec 6;14(12):e1007850. doi: 10.1371/journal.pgen.1007850 (PMC6303079; doi:10.1371/journal.pgen.1007850)
Supplement: S6 Table — (DOCX) [file pgen.1007850.s006.docx]

**S5 Table SMOC2 Line 1 insertion (CFA1:58,989,568) Genotyping**

| ***SMOC2* Line 1 insertion (CFA1:58,989,568) Genotyping** | | | | | |
| --- | --- | --- | --- | --- | --- |
| **Breed** | **Total Genotyped** | **WT** | **HET** | **MT** | **Brachycephalic** |
| Bernese Mountain Dog | 3 | 3 | 0 | 0 | N |
| Border Collie | 5 | 5 | 0 | 0 | N |
| Boston Terrier | 31 | 0 | 3 | 28 | Y |
| Boxer | 17 | 1 | 0 | 16 | Y |
| Brussels Griffon | 1 | 0 | 0 | 1 | Y |
| Bull Mastiff | 10 | 6 | 3 | 1 | Y |
| Bulldog | 15 | 0 | 0 | 15 | Y |
| Cane Corso | 5 | 5 | 0 | 0 | N |
| Cavalier King Charles S. | 10 | 10 | 0 | 0 | Y |
| Chihuahua | 4 | 4 | 0 | 0 | N |
| Chow Chow | 4 | 4 | 0 | 0 | N |
| Dachshund | 1 | 1 | 0 | 0 | N |
| French Bulldog | 11 | 0 | 0 | 11 | Y |
| Golden Retriever | 17 | 17 | 0 | 0 | N |
| Labrador Retriever | 1 | 1 | 0 | 0 | N |
| Lhasa Apso | 4 | 2 | 1 | 1 | Y |
| Newfoundland | 3 | 3 | 0 | 0 | N |
| Pekingese | 5 | 2 | 0 | 3 | Y |
| Pug | 4 | 0 | 0 | 4 | Y |
| Shih Tzu | 1 | 0 | 1 | 0 | Y |
