# Supplementary figures and images for: Whole genome variant association across 100 dogs identifies a frame shift mutation in DISHEVELLED 2 which contributes to Robinow-like syndrome in Bulldogs and related screw tail dog breeds
Source: PLoS Genet. 2018 Dec 6;14(12):e1007850. doi: 10.1371/journal.pgen.1007850 (PMC6303079; doi:10.1371/journal.pgen.1007850)

**S1 Fig Hierarchical clustering of Identity by state distance for all sequenced dogs**

**
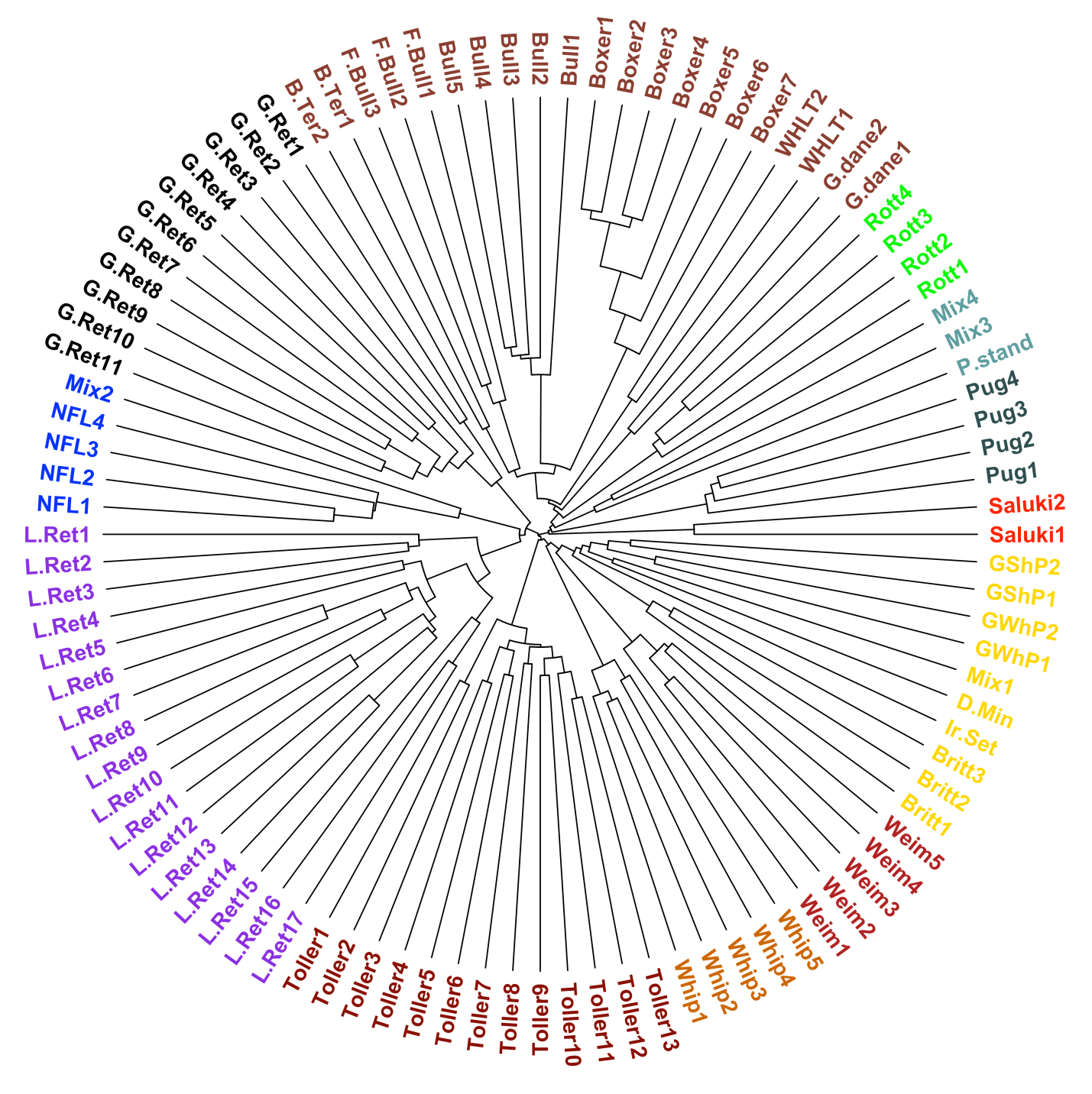
**

Supplement: S1 Fig — (DOCX) [file pgen.1007850.s007.docx]

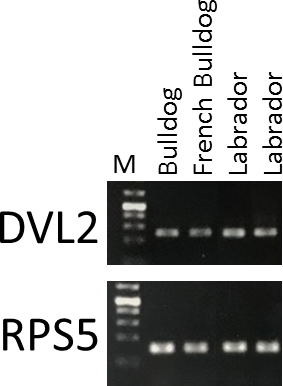

Supplement: S2 Fig — (TIF) [file pgen.1007850.s008.tif]

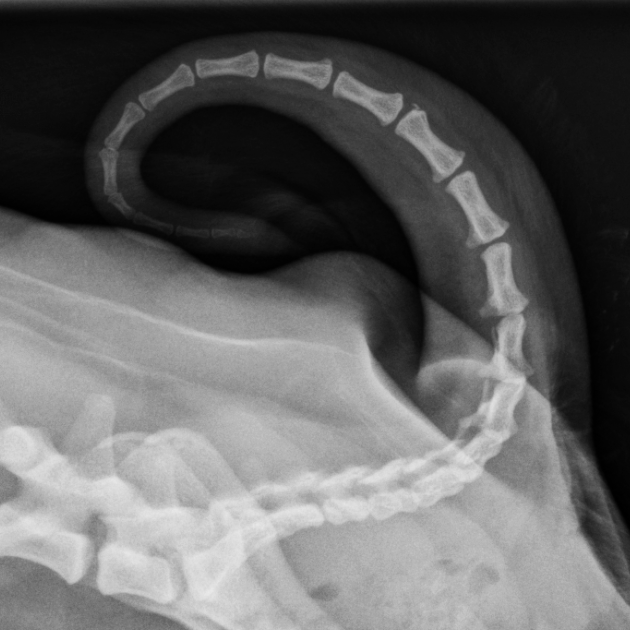

Supplement: S3 Fig — (TIF) [file pgen.1007850.s009.tif]
